# Wide-angle broadband antireflection coatings based on boomerang-like alumina nanostructures in visible region

**DOI:** 10.1038/s41598-022-04928-2

**Published:** 2022-01-18

**Authors:** MirKazem Omrani, Mohammad Malekmohammad, Hosein Zabolian

**Affiliations:** Department of Physics, University of Isfahan, P.O. Box 81746-7344, Isfahan, Iran

**Keywords:** Optical materials and structures, Sub-wavelength optics

## Abstract

A novel boomerang-like alumina based antireflective coating with ultra-low reflectance has been produced for light incidence angles form 0 up to 45°. Boomerang-like alumina nanostructures have been fabricated on the BK7 glass substrates by dip-coating and surface modification via hot water treatment. To achieve the lowest residual reflectance, the effect of dip-coating rate and hot-water temperature in the treatment process has been investigated and optimized. To further investigate the boomerang-like alumina nanostructure and extract its graded refractive index profile by fitting the measured reflectance spectrum with the simulated one, a simulation based on the finite-difference time-domain (FDTD) method has been performed. The average reflectance measured at normal incidence for double-sided coated BK7 glass substrates is only 0.3% in the visible spectral region. Considering both sides, the average reflectance of the substrate decreased in the spectral range of 400–700 nm down to 0.4% at incidence angles of 45° by applying the boomerang-like alumina antireflection coatings. The optimized single layer boomerang-like alumina coating on the curved aspheric lens exhibited a low average reflectance of less than 0.14% and an average transmittance of above 99.3% at normal incidence. The presented process is a simple and cost-effective route towards broadband and omnidirectional antireflection coatings, which have promising potential to be applied on substrates having large scales with complex geometric shapes.

## Introduction

The reflections of light that occurs at the interface between air and materials, due to the sharp change of refractive indices, must be eliminated in advanced optical systems to increase performance and reduce ghost images and flare. As a result, a single-layer anti-reflective coating must have a refractive index equal to the square root value of the substrate refractive index ($n_{coat} = \sqrt {n_{sub} }$) to achieve zero reflection, where the optical thickness of the anti-reflective layer is a quarter of the wavelength ($n_{coat} \times {\text{d}} = \frac{{\uplambda }}{4}$, where d is the physical thickness)^1,2^. For instance, an anti-reflective coating with a refractive index of less than 1.25 is required for fused silica substrates. Since there is no known material with this refractive index, only sub-wavelength nanoporous nanostructures, which are solid materials having air pores, can fulfill this requirement. However, in the case of meeting the above conditions, a zero reflectance is achieved solely at a specified wavelength due to the uniformity of the effective refractive index along the coating thickness. To achieve a broadband anti-reflective coating, the key issue is to avoid sharp changes in the effective refractive index between the air and the substrate. The refractive index of the coating should gradually increase from the coating surface to the interface. For this purpose, multilayer coatings have been developed in which the optical thickness of each layer is at least one-quarter of the wavelength and the refractive index of the layers from top to bottom should be incremental. According to optical theory, the refractive index of a multilayer stack must satisfy the equation: $\frac{{n_{1} }}{{n_{s} }} = \frac{{n_{2} }}{{n_{1} }} = \cdots = \frac{{n_{a} }}{{n_{k} }}$, where $n_{s}$ and $n_{a}$ are the reflective indices of the substrate and of air, respectively^3^.

On the other hand, modern optical systems are often based on lenses with different radii of curvature, and the incoming light impinges on the lens surface under various incidence angles. Therefore, providing an omnidirectional anti-reflective coating is of considerable importance. So far, the multifunctional broadband anti-reflective coatings are commonly prepared by physical vapor deposition (PVD) and chemical vapor deposition (CVD) techniques such as plasma-enhanced CVD, sputtering and evaporation^4–6^. However, thickness gradients can occur on curved substrate surfaces due to shadowing effects. Due to changes in film thickness, the coating in steep inclinations of the substrate surface does not meet the desired specification and leads to increased reflection^7^. Several methods have been proposed to solve this dilemma, in particular the use of shadowing masks and substrate-rotational systems^8–10^. However, these methods capabilities are limited to constrained geometric shapes, such as cylinders or convex lenses.

In recent years, sub-wavelength coatings with gradient refractive index profiles have been extensively studied in the hope of providing omnidirectional and broadband anti-reflective properties^1,11–15^. Moth’s eye nanostructures that provide gradual refractive index changes from the structure's surface to the interface minimize reflections well over a wide range of wavelengths and incident angles^16–23^. However, such nanostructures are usually prepared by photolithographic techniques, which are difficult to produce in large areas and on curved surfaces. Compared to these methods, the sol–gel method not only can be used in large substrates with complex shapes, but also has a controllable desirable microstructure to achieve low refractive index and hydrophobic performance, as well as low-cost simple operation process^24^.

To maximize light transmission, the coating nanostructure must be smaller than the wavelength of light to minimize scattering losses. Yamaguchi et al. have developed alumina-based flower-like structures with a roughness of less than 100 nm using the sol–gel method, which has a gradient density^25,26^. The average refractive index of the nanostructure in the specified depth gradually changes from the coating's surface to the interface, like a moth's eye structures. To create a gradient density in the alumina nano-porous structure, the coatings have been treated with hot water. They achieved a reflectance of less than 0.5% (1-side) in the visible spectral region by coating the soda-lime silica glass with flower-like alumina nanostructures using the dip-coating method^27^. However, as of yet, no study has been provided on the effect of dipping-withdrawing rate and hot water treatment temperature; the case which play a determinative role in the thickness and inclination of the effective refractive index changes on the optical response of sol–gel prepared alumina nanostructures.

Here, inspired by the manufacturing method of anti-reflective coatings based on flower-like alumina, in a sol–gel process, the boomerang-like alumina nanostructure has been coated on the BK7 glass substrate. The influence of hot-water treatment temperature and dipping-withdrawing rate has been investigated in order to achieve a high performance anti-reflective coating with high transparency. The omnidirectional property of the proposed coating due to having an inclined refractive index profile has been investigated and the optimized coating has been applied on curved aspheric lenses.

## Results and discussion

**Figure 1:**
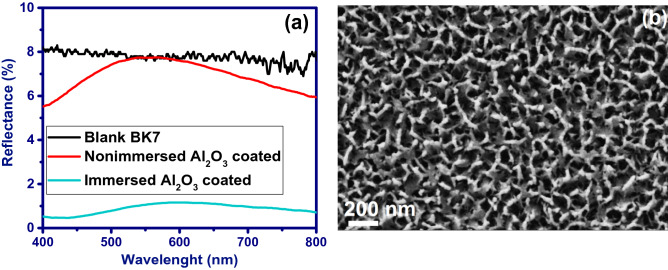
(**a**) Reflectance spectra measured from blank BK7 and double-side Al_2_O_3_ coated BK7 substrates with and without hot-water treatment. (**b**) Field emission scanning electron micrograph of the surface of Al_2_O_3_ thin film with boomerang-like structure.

Figure 1a shows the reflectance spectra of the blank BK7 substrate compared to the double-side porous alumina coated BK7 (dipping-withdrawing rate of 3 mm/s) in both the presence and absence of the hot-water treatment process. The low refractive index of porous alumina (~ 1.4) compared to BK7 substrate (~ 1.51) led to a decrease in light reflectance; the reduction which was significantly increased while the coated substrate was immersed in hot water. Hot-water treatment leads to the formation of boomerang-like structures with a roughness of less than 50 nm during the dissolution of porous Al_2_O_3_ film on its smooth and flat surface (Fig. 1b). This in turn leads to an increase in the density of alumina with an increasing distance from the coating's surface, creating a gradient refractive index profile, the same as a moth's eye nanostructure. However, the boomerang-like structure may not be perfectly formed along the coating thickness and there may be a gap of porous alumina between the boomerang-like structure and the substrate. Therefore, optimization of dipping-withdrawing rate and hot-water treatment temperature, which has a direct impact on the thickness and inclination of the refractive index profile, is important to achieve high anti-reflective properties.

**Figure 2:**
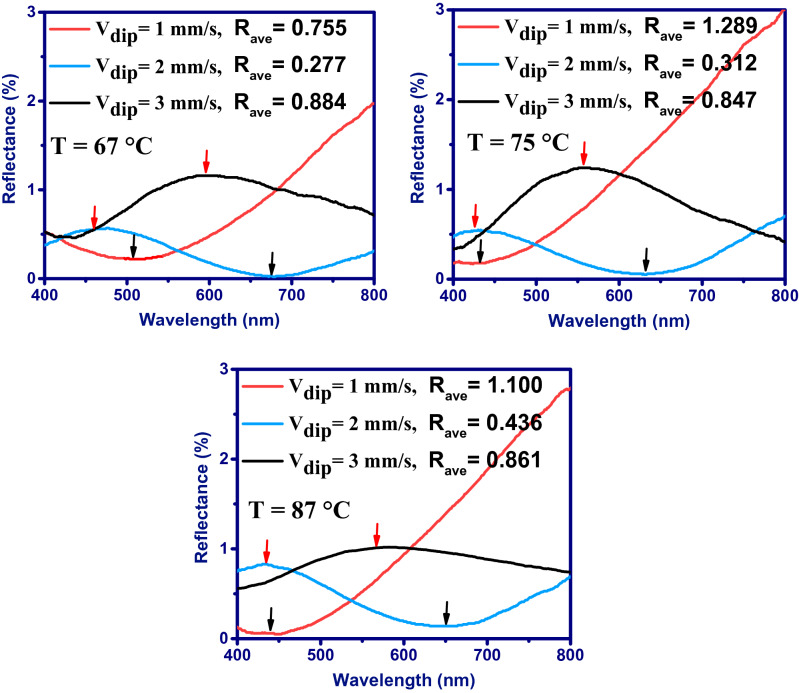
Reflectance spectra measured from double side coated BK7 substrates with boomerang-like Al_2_O_3_ films at different dipping-withdrawing rates (V_dip_) and hot-water treatment temperatures (T).

Figure 2 shows the hot-water treatment temperature effect on the optical response of double-side boomerang-like alumina coated BK7 substrates with different dipping-withdrawing rates (1, 2 and 3 mm/s). Previous studies have shown that high porosity occurs in the alumina layer at treatment temperatures above 60°C^25,26,28^. Therefore, for temperatures above 60 degrees, the effect of treatment temperature on the optical performance of alumina coating has been investigated. At all hot-water treatment temperatures of 67 °C, 75 °C and 87 °C, with increasing dipping-withdrawing rate from 1 to 2 mm/s, initially the reflectance decreases, then with increasing the rate up to 3 mm/s, the reflectance begin to increase. Among all these samples, the coatings prepared with a dipping-withdrawing rate of 2 mm/s (creates a thickness of about 200 nm^29^) shows the lowest light reflectance with average reflectance (R_ave_) of 0.277, 0.312 and 0.436% for hot-water treatment temperatures of 67 °C, 75 °C and 87 °C, respectively. Since the thickness of the coated layer is directly related to the dipping-withdrawing rate, increasing the rate causes the reflectance spectrum to have red-shifts as the layer thickness increases (as shown in Fig. 2). The blue-shift of the reflectance spectrum with increasing hot-water treatment temperature from 67 °C to 75 °C and 87 °C can be due to more dissolution of alumina and reduction of layer thickness. Excessive increase of this dissolution in the upper levels of alumina boomerang-like structure, which is more exposed to hot water, can lead to increased light reflectance by increasing the slope of the refractive index profile. For example, in the coating prepared with the rate of 2 mm/s, the light reflectance has increased over the entire visible spectral region due to increasing the treatment temperature from 75 °C to 87 °C, while the layer thickness has not significantly changed (inferred from this observation that there was no shift in the reflectance spectrum). Therefore, increasing light reflectance can be attributed to increasing the slope of the refractive index profile during high dissolution of the upper levels of the boomerang-like structure of alumina.

**Figure 3:**
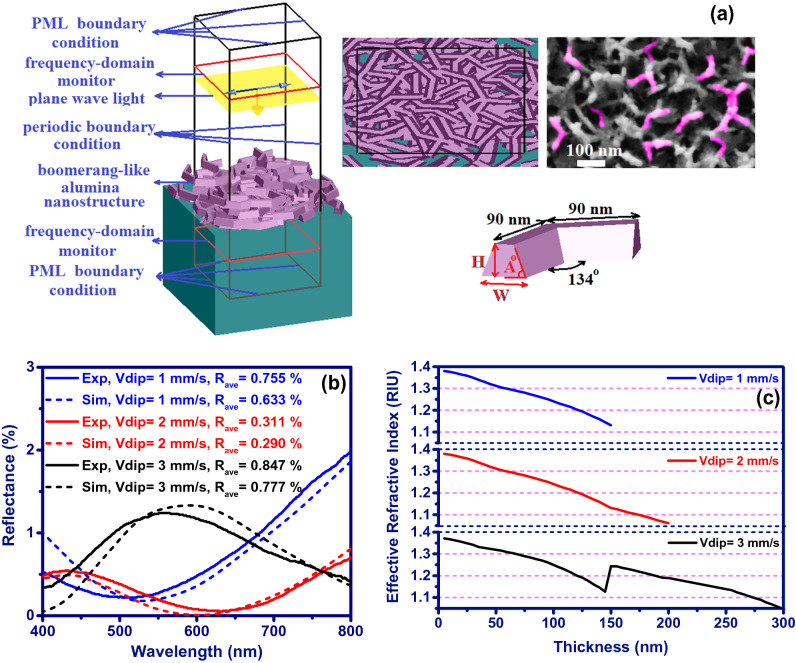
(**a**) Schematic diagram of the simulation model: perspective view (left) and top view (right). (**b**) Spectral reflectance of double side coated samples with different dipping-withdrawing rates (V_dip_) from Fig. 2 (solid line) and simulated spectral reflectance (dash line). (**c**) The relationships between thickness position and effective refractive index of boomerang-like Al_2_O_3_ films produced at different dipping-withdrawing rates (V_dip_).

To further investigate the boomerang-like alumina nanostructure and extract its refractive index profile by fitting the measured reflectance spectrum with the simulated one, a simulation based on the finite-difference time-domain (FDTD) method has been performed (Fig. 3). The boomerang-like unit structure, inspired by the FESEM image of the sample surface (Fig. 3a), has been used to design the alumina nanostructure. Nano-boomerangs with a height, H of 50 nm, a width, W of 30 and 40 nm, and a basic angle, A of 77–83 degrees have been randomly distributed (random position and rotation) on the surface of BK7 substrate so that thicknesses of 150, 200 and 300 nm with gradient refractive index profiles (Fig. 3c) have been created for the coatings fabricated at dipping-withdrawing rates of 1, 2 and 3 mm/s, respectively. Figure 3b shows the measured reflectance spectra of the fabricated anti-reflective coatings compared to the simulation results. The calculated reflectance spectrum is in good agreement with the experimental spectra, which shows that the modeling performed in this work can well describe the optical performance of the device. Figure 3c shows the variation of effective refractive index with thickness for coated alumina nanostructures at rates of 1, 2 and 3 mm/s. Among them, the boomerang-like alumina nanostructure coated at a rate of 2 mm/s and treated with deionized hot water for 30 min, which provides a gradient refractive index profile rated from 1.05 to 1.4 along the 200 nm thickness, has been presented the best optical performance with the least amount of reflectance (Fig. 3b). The effective refractive index along the alumina nanostructure has been calculated using Eq. (1) provided by Yoldas for porous nanostructures^30^.

1 $$ n_{p}^{2} = \left( {n^{2} - 1} \right)\left( {1 - \frac{p}{100}} \right) + 1, $$

where $p$, $n$, and $n_{p}$ are the percent porosity, index of nonporous material, and index of porous material, respectively. The percent porosity has been calculated using an index monitor along the nanostructure thickness and image processing.

**Figure 4:**
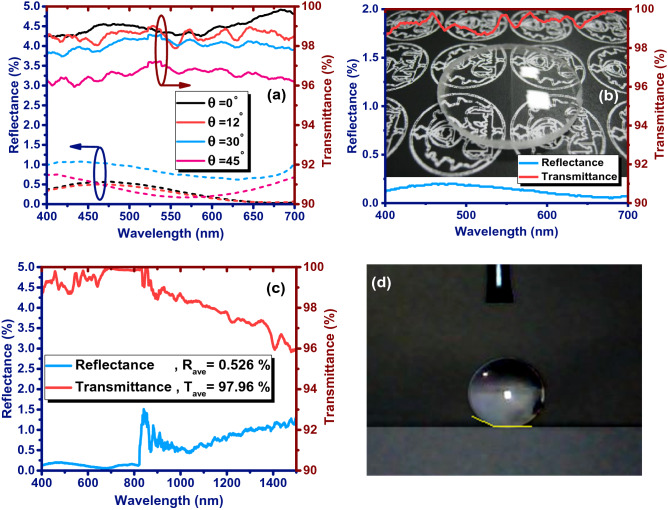
(**a**) Reflectance and transmittance spectra measured from double side coated BK7 substrates with boomerang-like Al_2_O_3_ films at incident angles of 0°, 12°, 30° and 45°. (**b**) Reflectance and transmittance across a boomerang-like alumina coated lens surface measured under light at normal incidence. The background image in (**b**) shows that the transparency of the BK7 lens (the right part of lens) enhances remarkably in the presence of boomerang-like alumina coating (the left part of lens). (**c**) Reflectance and transmittance across a boomerang-like alumina coated lens surface measured under light at normal incidence. (**d**) Photograph of water droplet on the superhydrophobic surfaces of boomerang-like alumina coated BK7 substrate.

Figure 4a shows the reflectance and transmittance spectra of double-side boomerang-like alumina coated BK7 substrates with the dipping-withdrawing rate of 2 mm/s and the hot-water treatment temperature of 67 °C at the incidence angles of 0°, 12°, 30° and 45°. At the case of normal incidence, the average reflectance of BK7 has reduced from about 8% to 0.3% in the visible spectral region. Measurements at all incidence angles show a low average reflectance of less than 0.9%. Similarly, the average transmittance at the incidence angle of 45° is above 96.5%, which is significantly higher than 92% for the blank BK7 at the normal incidence. Subsequently, the optimized boomerang-like alumina coating is applied to a curved aspheric BK7 lens and its anti-reflective performance is investigated (Fig. 4b). The measurements showed an average reflectance of less than 0.14% and an average transmittance of above 99.3% in the visible spectral region (400–700 nm). To realize the effect of broadband anti-reflection property in the visible wavelength range, optical images for a lens with and without the proposed coating that was exposed to white light are shown in the background of Fig. 4b. The right part of the lens (without the coating) looks a bit white and misty gray, and the image behind the lens cannot be clearly seen due to the ghost image from the light. In general, the uncoated part reflects all kinds of visible light, and the lens appears white. On the contrary, the left part of the lens (with the coating) shows increased transparency, and the real color of the background image is reproduced phenomenally even at oblique incidence angles.

It is noteworthy that this anti-reflective property of the proposed coating is not only restricted to the visible spectral region but also provide low reflectance (average of 0.526%) with very high transparency (average transmittance of 97.96%) in the wavelength range of 400–1500 nm (Fig. 4c). Above all, this nanostructure shows hydrophilic properties due to its roughness of less than 50 nm, which provides superhydrophobic properties during the reduction of surface free energy using fluorosilane (Fig. 4d). In the case of BK7 glass substrate coated with boomerang-like Al_2_O_3_ thin film, the contact angle for water drop is about 155°. Here, heptadecafluorodecyltrimethoxysilane, which is one of the fluoroalkyltrimethoxysilanes (FASs) was spin coated over boomerang-like alumina coatings at 3000 RPM for 60 s and annealed at 180 °C for 30 min to obtain low energy surfaces.

The various techniques used to fabricate alumina-based single-layer anti-reflective coatings have been summarized in Table 1. Lertvanithphol et al. have fabricated Al_2_O_3_ nanoflake films on a glass slide by reactive magnetron sputtering and surface modification via hot water treatment and alkali treatment^31^. The developed anti-reflective coating reduced the average reflectance (both sides) from 9.19% to 4.12%. Using a solution-based approach and spin coating technique, Sutha et al. have developed an amorphous alumina coating with an interconnected porous network of nanoflakes on a soda glass substrate^32^. The 300 nm thick coating produced an average reflectance of 3.04%. The hierarchical pseudo-boehmite nanoflakelets fabricated using custom-made wire bar coater on solar cover glass by Joghee et al. reduced light reflectance to 1.59%^33^. In another study, pseudo-boehmite nanocrystals formed on the surface of alumina gel by immersion in hot water, in which alumina was coated on the poly (methyl methacrylate) PMMA substrate using sol–gel process and dip-coating technique, decreased the average reflectance from 8.06% to 1.44%^26^. Using a similar manufacturing process, Yamaguchi et al. have produced flower-like alumina nanostructures on the soda glass substrate, which yielded an average reflectance of 1.16% in the visible region^25^. Uniquely, Kauppinen et al. have fabricated a grass-like alumina-based anti-reflective coating with a graded refractive index profile using atomic layer deposition (ALD) and the hot-water treatment process on soda glass. By investigating and optimizing the water temperature in the treatment process, they reduced the average reflectance from 8.09% to 0.77%^28^. Considering all of the above studies, we have used a cost-effective and simple sol–gel process in which boomerang-like alumina nanostructures have been fabricated on the BK7 substrate in the environment atmosphere without humidity control. This anti-reflective coating, which can be easily fabricated on aspheric lens using the proposed method, has achieved an average reflectance (both sides) of less than 0.5% (~ 0.31%).

**Table 1:** Comparison of various fabrication techniques used for Al_2_O_3_ based single-layer antireflection coatings in visible region (400–700 nm).

Substrate	Fabrication method	Al_2_O_3_ nanostructure	Coated (uncoated) average R (%)	Ref
Glass	Reactive magnetron sputtering	Nanoflake	4.12 (9.19)	^31^
Soda lime glass	Spin-coating	Nanoflakes	3.04 (7.29)	^32^
Solar cover glass	Wire bar coating	Nanoflakelet	1.59 (11.61)	^33^
PMMA	Dip-coating	Pseudo-boehmite nanocrystals	1.44 (8.06)	^26^
Soda lime glass	Dip-coating	Flowerlike	1.16 (9.76)	^25^
Soda lime glass	Atomic layer deposition	Grasslike	0.77 (8.09)	^28^
BK7 glass	Dip-coating	Boomeranglike	0.31 (7.85)	T.W

## Conclusion

In conclusion, an omnidirectional and broadband antireflection coating based on the boomerang-like alumina nanostructures prepared by dip-coating and hot-water treatment has been demonstrated. The effects of hot water treatment temperature and the rate of dip-coating, which determines the thickness of the anti-reflective coating, on its optical performance have been investigated. A low reflectance with an average reflectance of less than 0.3% was realized in the visible spectral region at normal incidence (double-side) for the boomerang-like alumina nanostructures fabricated with a rate of 2 mm/s (~ 200 nm) and a hot water treatment temperature of 67 °C. The omnidirectional properties of the developed coating are discussed for incidence angles ranging from 0 to 45°. The average reflectance for a double-side coated BK7 substrate has been less than 0.4% at 45° incidence angle. An average transmittance of above 99.3% with optical losses of 0.56% is achieved for a double-side coated curved aspheric BK7 lens. The proposed coating superiority is that the boomerang-like alumina nanostructure can be applied on inclined surfaces in a broad wavelength range because of having a gradient refractive index profile. In addition, the complete antireflection system can be coated in one process followed by one hot-water treatment.

## Methods

### Materials and preparation of Al_2_O_3_ sol

Aluminum-tri-sec-butoxide (Al(O-sec-Bu)_3_) was used as a starting material. Al(O-sec-Bu)_3_ and isopropyl alcohol (i-PrOH) were mixed and stirred at room temperature for 20 min. Ethylacetoacetate (EAcAc) was added to the solution as a chelating agent, and the solution was stirred for 20 min. Then, diluted deionized water with i-PrOH was added dropwise to the solution for hydrolysis process. The molar ratios of EAcAc, H_2_O and i-PrOH to Al(O-sec-Bu)_3_ were kept to be 1, 1 and 20, respectively^27^. All materials were obtained from Merck Chemicals.

### Film preparation and characterization

The coating was deposited on BK7 glasses via dip-coating manner (with various drawing speed) in an ambient atmosphere without the humidity control. The obtained coating were heat-treated at 400 °C for 30 min to get porous Al_2_O_3_ films^27,29^. Then the porous Al_2_O_3_ coatings were immersed in hot water (with various temperature) for 60 min and, after being dried in an ambient atmosphere, were heat-treated again at 400 °C for 30 min. UV–visible spectrophotometer (Shimadzu, UV-3100) was used to measure the transmittance and reflectance of the coatings. The microstructural details of the coatings were investigated using high-resolution field emission scanning electron microscopy (FESEM, model Sigma VP, Zeiss).
